# Supplementary material for: Chromothripsis during telomere crisis is independent of NHEJ, and consistent with a replicative origin
Source: Genome Res. 2019 May;29(5):737–49. doi: 10.1101/gr.240705.118 (PMC6499312; doi:10.1101/gr.240705.118)
Supplement: Supplemental Material [file supp_gr.240705.118_Supplemental_file_1.zip › contigs/annotated_contigs/DB38/contig.2.DB38_length_652_mean_cov_10.0460122699.docx]

**DB38_length_652_mean_cov_10.0460122699**

```
GAAGTGATGGGGAGAAACGTGGAAGAACTAAGAGAAAGAAAAGCAGCCGCTGACAGCCAGGAAATGGCCCGGACTGCCCACCGGGCCTT
 >chr2:17832680-17833008 - E=1e-185
GGTGCTGCCGGGAGCTGGCCCAGCCCTCACAGTCAGGTCTCACACCAAACATAACCAATGTCACAAAATGCCAGTGACCATGATGGAGC

AAGGCAAAAACAAGACCACTCTGTCATTAAGTCTGAACACAGACACAAAACACCAAGCATCCCTGCTTCCTGCTATGGTGAGTGACCAC

TGCTTCTTCACCCGTTAAGAGCATTGCATTGGTCTGTGTGCCCTCCTCCTAGGTATCTA|AT|TCTTGTCGAAGGTTCGGAAGGCATGC
                                                            >chr2:17830413-17830739 - E
TGGGCAAACTTGGAGGCGTCTCCATAAGGAAAGAACTGCAAAGGAGAACACGCGCAGTCAGGTGTCGGGAGGGGATGGAATCGGGGCGA
=2e-184
CGAGGTAGGTTCCTAATGACGGCATCACAGGTGCTCCTGGGCGTTGTCGGAGCCTCACCCATTGGAGAGCAGTTTGGCCTGACTGTCAA

GGGCAAGGGCAGGCATTAGGGGAGGCTTTCTCACAGCAGGTGCTCAGGTACAGGCATCTCATTGTTTCCTGTGGGTGAGGCTGGTGACA

TGTGGCTTTCAGTTGATGGGGCTTGGAATCG
```
